# Very Late-Onset Myasthenia Gravis in a Very Elderly Patient: Diagnostic Challenges and Importance of Early Recognition

**DOI:** 10.3390/geriatrics11050121

**Published:** 2026-09-04

**Authors:** Nermina Polimac Gorana, Almedina Spiljak

**Affiliations:** 1 Department of Neurology, General Hospital “Prim. dr. Abdulah Nakaš”, 71000 Sarajevo, Bosnia and Herzegovina; 2 General Practice, Institute for Healthcare of Transportation Employees Sarajevo, 71000 Sarajevo, Bosnia and Herzegovina

**Keywords:** myasthenia gravis, late-onset myasthenia gravis, very late-onset myasthenia gravis, case report

## Abstract

**Background:** Myasthenia gravis is an autoimmune disorder of the neuromuscular junction characterized by fluctuating skeletal muscle weakness. Late-onset MG (onset ≥ 50 and <65 years) and very late-onset MG (VLOMG; onset ≥ 65 years) are increasingly recognized subgroups, and diagnosis in very elderly patients remains challenging because symptoms frequently overlap with age-related conditions and comorbidities. **Case Presentation:** An 88-year-old man with very late-onset myasthenia gravis (symptom onset at approximately age 85) was urgently referred because of a several-day history of rapidly worsening dysphagia and dysarthria, superimposed on fluctuating diplopia, dysphagia, dysarthria, and fatigable bulbar symptoms that had progressively worsened over the preceding three years. Initial diagnostic evaluation was challenging because of advanced age, previous lacunar infarctions, and multiple comorbidities, including pulmonary thromboembolism, chronic kidney disease, and permanent pacemaker implantation (which precluded brain MRI). Neurological examination and the characteristic fluctuation of symptoms raised suspicion of myasthenia gravis. Serological testing confirmed markedly elevated acetylcholine receptor antibodies, whereas MuSK antibodies were negative. Repetitive nerve stimulation was not performed given the high antibody titer and unambiguous clinical presentation. Thoracic computed tomography excluded thymoma. Treatment with pyridostigmine, azathioprine (maintenance dose kept lower than standard due to chronic kidney disease stage IIIB), and low-dose prednisone (selected due to age and comorbidity profile) resulted in early, patient-reported clinical improvement (approximately 60% in speech and swallowing) over eight weeks of follow-up; a validated severity scale (MG-ADL) showed a score of six (scoring range 0–24). **Conclusions:** Myasthenia gravis should remain an important differential diagnosis in very elderly patients presenting with fluctuating ocular and bulbar symptoms, even in the presence of multiple comorbidities that may obscure the diagnosis. In this patient, early recognition, antibody testing, and individualised initiation of therapy were followed by meaningful short-term improvement; a single case with eight weeks of follow-up cannot establish that such therapy prevents disease progression or myasthenic crisis, and longer follow-up and additional cases are needed.

## 1. Background

Myasthenia gravis (MG) is an autoimmune disease of the neuromuscular junction characterized by fluctuating, fatigable skeletal muscle weakness resulting from autoantibody-mediated impairment of postsynaptic neuromuscular transmission, most commonly targeting the acetylcholine receptor (AChR). MG is primarily mediated by autoantibodies directed against components of the postsynaptic membrane, leading to impaired neuromuscular transmission and characteristic fatigable muscle weakness. Clinical manifestations are heterogeneous and may involve ocular, bulbar, respiratory, axial, and limb muscles. Ocular symptoms are often the initial presentation, whereas generalized weakness may develop as the disease progresses [1,2].

The diagnosis of myasthenia gravis is based on a combination of characteristic clinical features, serological testing, and neurophysiological studies. Detection of acetylcholine receptor (AChR) antibodies is the first-line serological investigation, while muscle-specific tyrosine kinase (MuSK) antibodies should be assessed in seronegative patients. Repetitive nerve stimulation and single-fibre electromyography may further support the diagnosis when indicated, particularly in seronegative cases or when the clinical picture is ambiguous. In addition, thoracic imaging is recommended in all patients to exclude thymoma [3].

Treatment is individualized according to disease severity and clinical presentation. Acetylcholinesterase inhibitors, particularly pyridostigmine, are recommended as first-line symptomatic therapy, whereas corticosteroids and other immunosuppressive agents are used in patients with persistent or generalized disease. Intravenous immunoglobulin or plasma exchange is reserved for severe exacerbations or myasthenic crisis [3].

Age-based nomenclature varies across the literature, and this is directly relevant to the present case. We follow the classification proposed by Cortés-Vicente et al., in which early onset MG denotes onset before age 50, late-onset MG denotes onset ≥ 50 and <65 years, and very late-onset MG (VLOMG) denotes onset ≥ 65 years. Because our patient’s symptoms began at approximately age 85, his disease is more precisely classified as VLOMG rather than late-onset MG, and we use this term consistently throughout the report. His presentation shares several features previously reported as characteristic of VLOMG: male sex, predominant ocular/bulbar onset, AChR-antibody seropositivity, MuSK-antibody negativity, and absence of thymoma [4,5].

Late-onset and very late-onset myasthenia gravis are increasingly recognized in the aging population. Nevertheless, diagnosis remains challenging, as symptoms are often attributed to aging, frailty, or coexisting neurological disorders, resulting in delayed recognition and occasional misdiagnosis [6]. Recently published case reports of very late-onset myasthenia gravis have similarly described elderly patients presenting predominantly with bulbar or ocular symptoms, positive anti-AChR antibodies, and no evidence of thymoma on thoracic imaging, at times closely mimicking cerebrovascular disease [7,8,9,10]. Taken together, these findings underscore that myasthenia gravis should remain an important differential diagnosis in very elderly patients presenting with fluctuating ocular or bulbar symptoms or unexplained muscle weakness, and that early recognition, appropriate serological testing, and timely treatment are essential to improve clinical outcomes and avoid diagnostic delay.

We present the case of an 88-year-old patient with seropositive very late-onset myasthenia gravis (symptom onset at approximately age 85), highlighting the diagnostic challenges and the importance of timely recognition and appropriate treatment in very elderly patients.

## 2. Case Presentation

An 88-year-old man presented to his primary care physician accompanied by his wife because of a several-day history of progressively worsening dysphagia and dysarthria. According to his wife, the symptoms were mild in the morning but consistently worsened throughout the day, suggesting a fluctuating pattern of muscle weakness.

His past medical history included pulmonary thromboembolism treated with rivaroxaban, permanent pacemaker implantation, chronic kidney disease stage IIIB, hypertension, and anemia. Based on the characteristic fluctuating bulbar symptoms and the clinical suspicion of myasthenia gravis, the patient was urgently referred for neurological evaluation.

Further history revealed that approximately three years before the current presentation, the patient had developed intermittent vertigo, followed by persistent binocular diplopia in all directions of gaze, dysphagia, and subsequently dysarthria. The considerable delay between symptom onset and diagnosis likely reflected the tendency to attribute these complaints to cerebrovascular disease and age-related decline, underscoring the importance of maintaining a high index of suspicion for myasthenia gravis even when alternative explanations appear plausible. He reported that symptoms were consistently less pronounced in the morning and progressively worsened throughout the day with physical activity and fatigue. Previous brain computed tomography demonstrated lacunar infarctions; brain magnetic resonance imaging could not be performed because of the implanted permanent pacemaker.

### 2.1. Timeline of Symptom Onset, Evaluation, and Treatment

To make the diagnostic delay—the central message of this report—transparent, the sequence of events is summarised in Table 1.

Neurological examination demonstrated binocular diplopia in all directions of gaze, very mild bilateral ptosis, mild dysarthria that became more pronounced during sustained speech, and bulbar symptoms without significant limb weakness. Motor strength, muscle stretch reflexes, coordination, and sensory examination were otherwise preserved, while gait was mildly impaired due to previous bilateral hip surgery and shoulder osteoarthritis. The ice pack test was not performed as bilateral ptosis was very mild and the diagnosis was pursued serologically given the classical clinical picture.

### 2.2. Differential Diagnosis and Diagnostic Reasoning

Given the three-year history of diplopia, dysphagia, and dysarthria in a patient with known lacunar infarctions, cerebrovascular disease was the principal alternative diagnosis considered, and was in fact the explanation initially favoured. Several features argued against a vascular cause and in favour of MG: the marked diurnal and activity-related fluctuation of symptoms (improving with rest, worsening with sustained effort), the absence of other fixed focal neurological deficits referable to the previously documented lacunar infarcts, and the lack of a new acute vascular event on this presentation. Other causes of subacute diplopia, dysphagia, and dysarthria in an elderly patient—including brainstem structural lesions, bulbar-onset motor neuron disease, cranial neuropathies, thyroid eye disease, and other neuromuscular junction disorders such as Lambert–Eaton myasthenic syndrome—were considered less likely given the preserved reflexes, absence of upper or lower motor neuron signs outside the bulbar/ocular distribution, and the classic fatigable pattern. The markedly elevated AChR-antibody titer, in the context of this clinical picture, was considered sufficiently specific to confirm the diagnosis without further neurophysiological testing, consistent with current guidance [3].

Brain MRI, which can help exclude brainstem structural lesions, could not be performed because of the permanent pacemaker; this is an acknowledged limitation of the diagnostic work-up, and the exclusion of a brainstem lesion therefore rested on the non-progressive appearance of the previously documented lacunar infarcts on CT, the fluctuating/fatigable nature of the symptoms, and the subsequent antibody-confirmed diagnosis, rather than on contemporaneous cross-sectional imaging of the brainstem.

### 2.3. Quantification of Symptom Severity (MG-ADL)

To quantify symptom severity in a standardized way, we have mapped the documented clinical findings onto the eight-item Myasthenia Gravis Activities of Daily Living (MG-ADL) scale (each item scored 0 = normal to 3 = most severe) (Table 2).

### 2.4. Treatment Initiation, Monitoring, and Safety

Because of the characteristic fluctuation of ocular and bulbar symptoms, myasthenia gravis was suspected. Serological testing demonstrated markedly elevated acetylcholine receptor antibodies (>8.4 nmol/L; reference ≤ 0.45 nmol/L), whereas muscle-specific tyrosine kinase (MuSK) antibodies were negative, confirming seropositive acetylcholine receptor antibody-positive myasthenia gravis given the high antibody titer and unambiguous clinical presentation.

Treatment was initiated with pyridostigmine 60 mg once daily (chosen as the standard first-line symptomatic starting dose), which was titrated to 60 mg twice daily after 7 days. Azathioprine was subsequently introduced at 50 mg once daily and increased to 50 mg twice daily after 2 weeks; this cautious up-titration toward a lower-than-standard maintenance dose was deemed appropriate given the presence of stage IIIB chronic kidney disease. Prednisone was started at alternating doses of 5 mg and 10 mg on consecutive days during the first week of treatment. Owing to the patient’s advanced age and significant comorbidity burden, a cautious corticosteroid initiation strategy was adopted, with plans for progressive up-titration based on clinical efficacy and tolerability. Previous medications were continued.

Baseline safety assessment before starting azathioprine and prednisone was performed (full blood count, liver and kidney function tests, glucose, and blood pressure), and subsequent laboratory, adverse event, and medication adherence monitoring over the follow-up period was performed at regular intervals throughout treatment; no adverse drug reactions were observed, and the patient was adherent to the prescribed regimen. Given the patient’s stage IIIB chronic kidney disease and advanced age, explicit documentation of this monitoring is important to support the safety of the regimen described.

At follow-up eight weeks later, the patient reported approximately 60% improvement in speech and swallowing, although mild residual symptoms persisted. MG-ADL was 6 (baseline 12, scoring range 0–24).

Thoracic computed tomography performed to exclude thymoma demonstrated no mediastinal mass or thymic enlargement. Chronic pulmonary changes and residual findings consistent with previous pulmonary thromboembolism were noted incidentally.

Titin autoantibody testing could not be performed due to reagent unavailability.

Patient perspective: The patient and his wife expressed satisfaction with the diagnostic process and reported clinical improvement following treatment.

## 3. Conclusions

Very late-onset myasthenia gravis remains an important diagnostic challenge in very elderly patients, in whom fluctuating ocular and bulbar symptoms may easily be misattributed to aging, cerebrovascular disease, or other coexisting conditions. This case illustrates that a three-year diagnostic delay occurred precisely because symptoms were initially ascribed to lacunar infarctions and age-related frailty. Careful attention to the characteristic diurnal fluctuation and fatigability of symptoms, combined with targeted serological testing, ultimately enabled diagnosis despite multiple potential confounders—this diagnostic lesson, rather than any conclusion about treatment effectiveness, is the principal contribution of this report.

This case also illustrates the practical challenge of tailoring treatment to patient-specific factors. The reduced azathioprine dose reflected impaired renal function, while the intentionally low prednisone dose was chosen to minimize adverse effects in an elderly patient with multiple comorbidities, with the expectation of gradual up-titration. Over eight weeks of follow-up, and alongside continuation of the patient’s other medications, meaningful short-term, patient-reported improvement was observed; this single, short case course does not by itself demonstrate the effectiveness or safety of this regimen beyond this individual patient, and no conclusions about its generalisability should be drawn from it.

Acetylcholine receptor antibody-positive very late-onset myasthenia gravis should remain an essential differential diagnosis in elderly patients presenting with unexplained diplopia, dysphagia, dysarthria, or fatigable weakness. Advanced age should never preclude consideration of this diagnosis. With an aging population and the increasing recognition of very late-onset myasthenia gravis, maintaining a high index of suspicion is essential to avoid diagnostic delay and to support timely, individualised treatment; whether such treatment reliably prevents myasthenic crisis cannot be concluded from a single case and would require longer follow-up and larger case series.

## Figures and Tables

**Table 1:** Timeline of symptom onset, evaluation and treatment.

Time Point	Event
~3 years before referral	Intermittent vertigo, followed by binocular diplopia in all directions of gaze; symptoms fluctuating, worse later in the day (first neurology consultation 2023).
Course of the 3-year interval	Progressive addition of dysphagia and then dysarthria; symptoms attributed to prior lacunar infarcts/age-related decline (neurological follow-up, 2025—with emphasis on cardiology, orthopedic, and nephrology monitoring).
Several days before referral	Acute-on-chronic worsening of dysphagia and dysarthria, diurnal fluctuation (mild in the morning, worse through the day), prompting the patient’s wife to seek urgent primary-care review.
Day 0	Primary-care assessment; clinical suspicion of MG raised on the basis of fluctuating bulbar symptoms; urgent neurology referral.
Neurology evaluation	History and examination as described below; AChR-antibody and MuSK-antibody testing sent; ice-pack test not performed (diagnosis pursued serologically).
Month 1	Follow-up—AChR-antibody result reported: markedly elevated (>8.4 nmol/L; reference ≤ 0.45 nmol/L); MuSK antibody negative.
Month 2	Follow-up—Thoracic CT performed—no mediastinal mass or thymic enlargement; incidental chronic post-embolic changes.
Treatment day 1	Pyridostigmine 60 mg once daily started.
Treatment day 7	Pyridostigmine increased to 60 mg twice daily.
Treatment day 7	Azathioprine 50 mg once daily started.
2 weeks after azathioprine start	Azathioprine increased to 50 mg twice daily.
Treatment week 1	Prednisone started at alternating 5 mg/10 mg on consecutive days.
5 weeks after treatment initiation	Follow-up visit: patient-reported ~40% improvement in speech and swallowing; MG-ADL- 8 (scoring range 0/24).
8 weeks after treatment initiation	Follow-up visit: patient-reported ~60% improvement in speech and swallowing; MG-ADL- 6 (scoring range 0/24).

**Table 2:** Myasthenia Gravis Activities of Daily Living scale/baseline, 8 weeks follow up.

MG-ADL Item	Baseline (Pre-Treatment)	8-Week Follow-Up
Talking	Documented dysarthria, worse with sustained speech assign 3	~60% patient-reported improvement assign 1
Chewing	assign 1	assign 0
Swallowing	assign 2	~60% patient-reported improvement assign 1
Breathing	assign 0	assign 0
Ability to brush teeth or comb hair	assign 1	assign 1
Ability to arise from a chair	assign 1	assign 0
Double vision (diplopia)	assign 3	assign 3
Eyelid droop (ptosis)	assign 1	assign 0

## Data Availability

All data relevant to this case are included in the article. Further information is available from the corresponding author upon reasonable request.

## Abbreviations

The following abbreviations are used in this manuscript:

AChR	acetylcholine receptor
MG	myasthenia gravis
MG-ADL	Myasthenia Gravis Activities of Daily Living scale
MuSK	muscle-specific tyrosine kinase
VLOMG	very late-onset myasthenia gravis
